# *Dragonfly*: an implementation of the expand–maximize–compress algorithm for single-particle imaging^1^

**DOI:** 10.1107/S1600576716008165

**Published:** 2016-06-20

**Authors:** Kartik Ayyer, Ti-Yen Lan, Veit Elser, N. Duane Loh

**Affiliations:** a Center for Free-Electron Laser Science, Deutsches Elektronen-Synchrotron DESY, Notkestrasse 85, 22607 Hamburg, Germany; b Laboratory of Atomic and Solid State Physics, Cornell University, Ithaca, NY 14853, USA; c Centre for Bio-imaging Sciences, National University of Singapore, 14 Science Drive 4, 117557, Singapore; d Department of Physics, National University of Singapore, 2 Science Drive 3, 117551, Singapore; e Department of Biological Sciences, National University of Singapore, 14 Science Drive 4, 117557, Singapore

**Keywords:** single-particle imaging, X-ray free-electron lasers, XFELs, expand–maximize–compress reconstruction algorithm

## Abstract

Single-particle imaging (SPI) with X-ray free-electron lasers has the potential to change fundamentally how biomacromolecules are imaged. The structure would be derived from millions of diffraction patterns, each from a different copy of the macromolecule before it is torn apart by radiation damage. The challenges posed by the resultant data stream are staggering: millions of incomplete, noisy and un-oriented patterns have to be computationally assembled into a three-dimensional intensity map and then phase reconstructed. In this paper, the *Dragonfly* software package is described, based on a parallel implementation of the expand–maximize–compress reconstruction algorithm that is well suited for this task. Auxiliary modules to simulate SPI data streams are also included to assess the feasibility of proposed SPI experiments at the Linac Coherent Light Source, Stanford, California, USA.

## Abstract 2

A description is given of a single-particle X-ray imaging reconstruction and simulation package using the expand–maximize–compress algorithm, named *Dragonfly*.

## 1. Introduction   

The Single-Particle Imaging Initiative (Aquila *et al.*, 2015 ▸) at the Linac Coherent Light Source (LCLS; Stanford, California, USA) is working towards single-particle imaging (SPI) of large biomolecules to 3 Å resolution. To prepare for a future where SPI is routine, we are making available a software package that will make this new imaging modality accessible to a broad user base.

The defining characteristics of an SPI experiment are now well known (Neutze *et al.*, 2000 ▸): the aim is to collect individual noisy diffraction patterns from very many reasonably identical copies of a particle, injected with unknown orientations into a pulsed X-ray beam. The expand–maximize–compress (EMC) algorithm (Loh & Elser, 2009 ▸) was developed specifically for processing SPI data sets. It was designed to take advantage of all the available information in these experiments, while also scaling well computationally. To obtain a better sense of the information-processing advantages of EMC, we briefly contrast it with two alternative methods that have been proposed.

Manifold embedding methods (Fung *et al.*, 2008 ▸; Schwander *et al.*, 2012 ▸) try to find a consistent set of particle orientations by identifying pairs of similar diffraction patterns that establish an adjacency network for embedding into the space of orientations. Nowhere does this method impose consistency between the many more pairs of diffraction patterns that are not similar. By contrast, EMC imposes consistency between each diffraction pattern and a three-dimensional intensity model built from a tentative orientation reconstruction of all the patterns.

Intensity cross-correlation methods offer another approach for deriving structure from un-oriented particle ensembles (Kam, 1977 ▸; Saldin *et al.*, 2010 ▸; Donatelli *et al.*, 2015 ▸). These methods work best when the X-ray flux passes through the fewest number of particles. However, in the single-particle limit these methods work at an enormous information deficit relative to EMC. This is because EMC uses the correlated arrangement of all 100–1000 photons in a typical diffraction pattern, rather than just the correlations between pairs.

While EMC is just beginning to be used for SPI of bio­particles, it has been field-tested in a number of proof-of-principle experiments (Loh *et al.*, 2010 ▸; Philipp *et al.*, 2012 ▸; Ayyer *et al.*, 2014 ▸; Ayyer, Philipp *et al.*, 2015 ▸; Ekeberg *et al.*, 2015 ▸; Wierman *et al.*, 2016 ▸). The most significant feature of these experiments is the demonstration that EMC’s probabilistic modeling of the detector photon counts continues to be valid even when the counts per scattering pattern are extremely sparse. Recording highly sparse data, with the hope that they reveal structure, will require a leap of faith on the part of structural biologists. Our EMC-based software package comes with tools to make that leap less blind for new users.

## 2. Purpose and structure of *Dragonfly*   

This software package was named *Dragonfly*, since the compound eyes of a dragonfly allow it a wide field of view and reputedly good vision for catching prey. It uses the EMC algorithm to reconstruct a three-dimensional diffraction volume from noisy randomly oriented SPI diffraction patterns. These patterns could be from simulations or actual SPI experiments. Although this package includes a data-stream generator that feeds simulated data into the EMC reconstruction algorithm, the algorithm can also take data from physical experiments, as long as the input/output formats specified here are used.

### 2.1. Key parameters in single-particle imaging   

**Figure 1:**
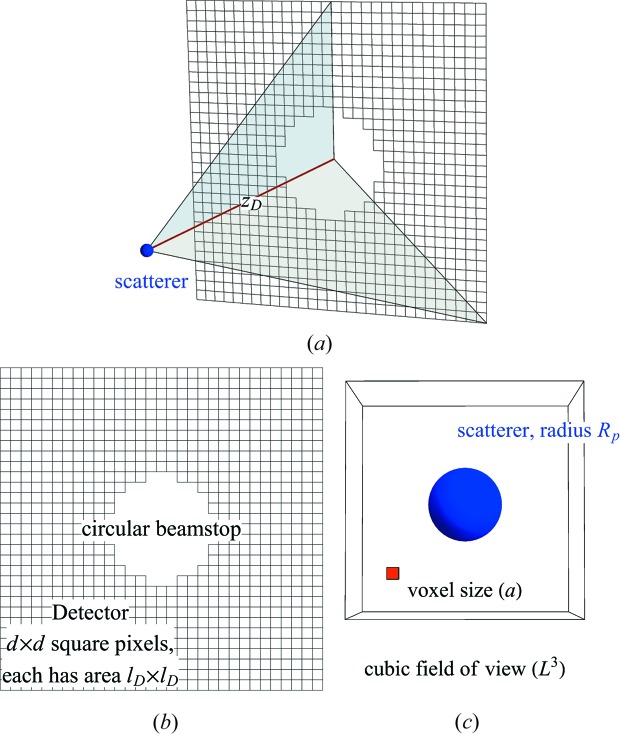
(*a*) The experimental geometry of single-particle imaging adopted in the data-stream simulator. (*b*) This simulator implements a planar square detector comprising *d* × *d* square pixels, each of area 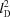. The detector is positioned at *z*_D_ from the X-ray interaction region, where (*c*) the scatterer (depicted here as a sphere of radius *R_p_*) is typically an electron-density map sampled from a Protein Data Bank file. From these, one can compute the maximum scattering angle captured by the detector, subtended by grey triangles in part (*a*) to either the edge or corner of the detector. Here, we take this maximum angle φ_max_ as the latter. Combined with the incident photon wavelength λ, this allows us to determine the half-period resolution, *a*, from the detector’s edge, which is equivalent to the length of the voxel (red) in the reconstructed electron-density map.

The key parameters of an SPI experiment are illustrated in Fig. 1 ▸. They include the photon wavelength λ and the maximum scattering angle φ_max_. These parameters determine the half-period resolution *a* of the reconstructed electron-density map. Together with the beam fluence, these parameters can help one decide if a candidate scatterer can yield enough diffraction signal to the desired resolution. These parameters are revisited in §2.5.1.

Throughout this document, we adopt the crystallographers’ convention for the spatial frequency: 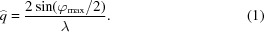
A corrective factor is applied to compensate for different solid angles subtended by different pixels on the detector (Appendix *B* [App appb]).

### 2.2. Reconstruction workflows in *Dragonfly*   

**Figure 2:**
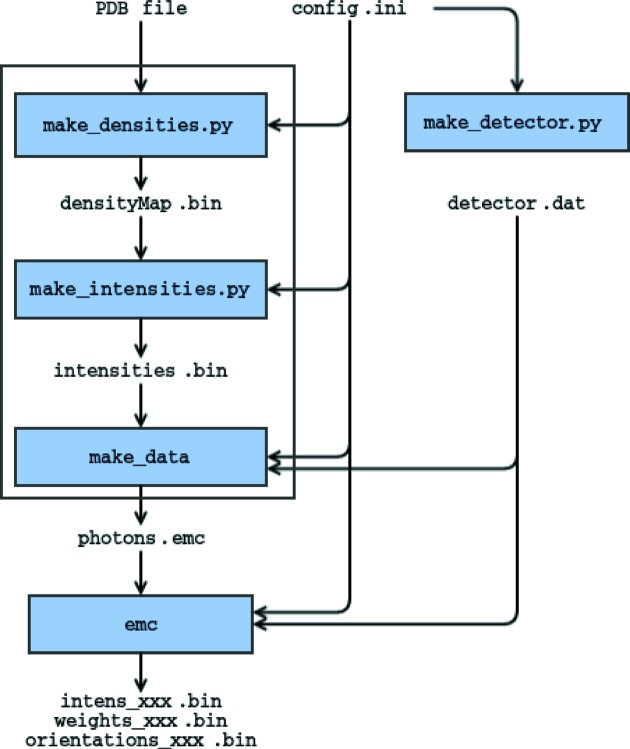
*Dragonfly* flowchart to simulate a data set and perform a reconstruction starting from a sample PDB file and a configuration file, config.ini, with information about the experimental setup. Input and output are shown as text, and modules as blue boxes. The large white rectangle defines the data-stream simulator.

**Figure 3:**
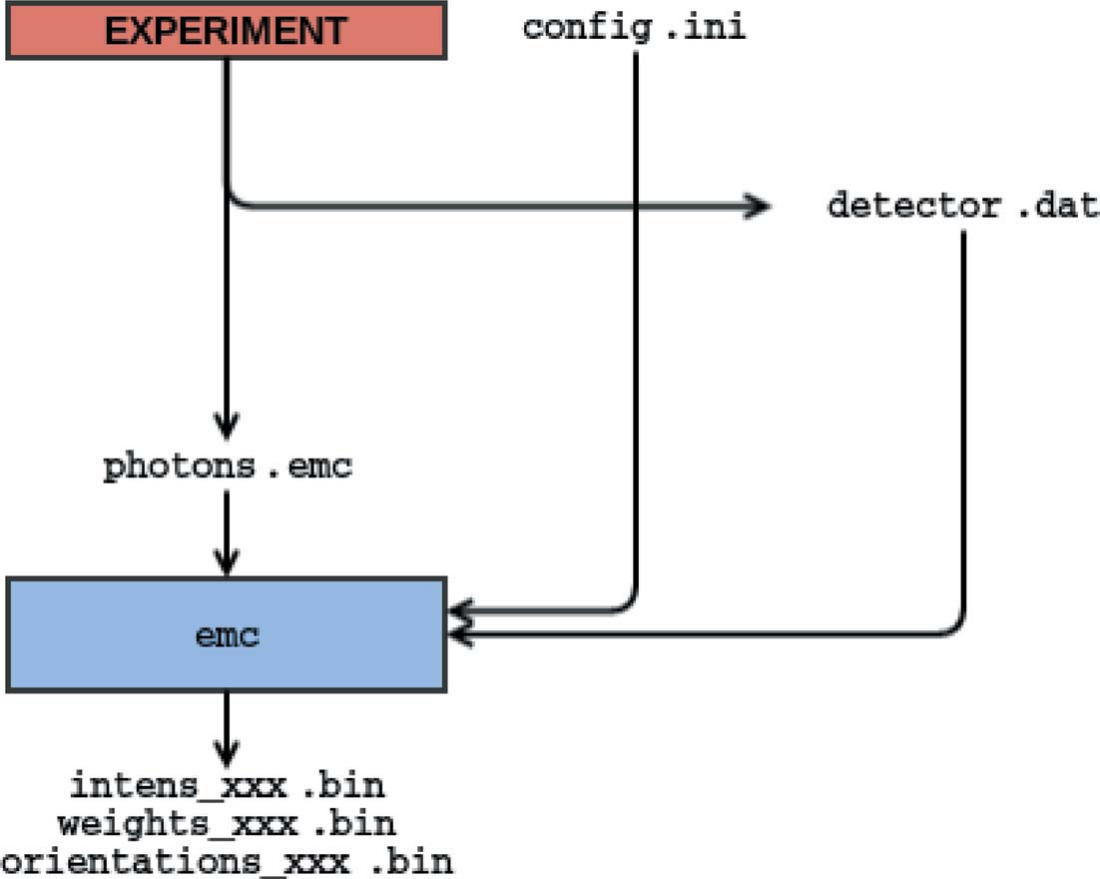
*Dragonfly* flowchart to process experimental data in sparse format. Information about the experimental parameters is placed in the configuration file config.ini and the detector geometry is in detector.dat. The formats of all three input files are described in §2.5. Notice that the difference between this workflow and that shown in Fig. 2 ▸ is in how the data are generated.

Whether the diffraction patterns are derived from simulations (Fig. 2 ▸) or experiments (Fig. 3 ▸), the minimum inputs to *Dragonfly* are a configuration file, a file containing detector coordinates plus pixel status, and a sparse representation of the photon data from diffraction patterns.

Modules and utilities within the *Dragonfly* package can be replaced by alternatives with compatible input and output data formats with other modules. In this package, binary files have extensions *.bin or *.emc; plain text files terminate with *.log, *.dat or *.ini.

### 2.3. Implementing the EMC algorithm   

The EMC algorithm (Loh & Elser, 2009 ▸) is an iterative reconstruction algorithm. It is implemented here with hybrid MPI+OpenMP (message passing interface + open multi-processing), and hence is suitable for both shared and distributed memory systems. In this section, we describe this implementation and an extension to deal with high-signal data.

In the current version, the code assumes a Poisson probability model for the number of photons in a pixel. Gaussian noise models have been used in situations with bright but noisy data (Loh *et al.*, 2010 ▸; Ekeberg *et al.*, 2015 ▸), but if single photons can be accurately counted, the noise model will be Poissonian.

We consider the Poisson noise model for a set of three-dimensional intensities *W*. Let the number of photons at pixel number *t* in a two-dimensional data frame (interchangeably termed photon/diffraction pattern) *d* be *K_dt_*, and for a given orientation *r* the predicted mean intensity at the same pixel be *W_rt_*. Since an independent Poisson process occurs at each pixel, the probability of that pattern being generated by a tomogram *W_r_* is 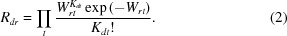
But, since the particle must have some orientation, the probability of frame *d* having orientation *r* is obtained by normalizing over all orientations:
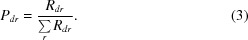
With these probabilities, one can define the model log-likelihood as the expectation of the total log-likelihood of the data being generated by a new model 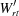: 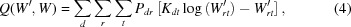
neglecting a model-independent constant. Maximizing *Q* with respect to the new model intensities 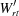 gives us the update rule 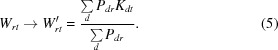
The most time-consuming step of each iteration is the calculation of equation (2). This involves comparing all the tomograms with all the patterns for each pixel which has at least one photon. The code is parallelized over orientations, so each MPI and OpenMP rank performs the calculation for a subset of orientations. At the start of the iterations, each MPI rank gets a copy of the current three-dimensional intensity model *W*. Each MPI and OpenMP rank then calculates the relevant tomograms, *W_rt_*, as needed and then computes *R_dr_* for that orientation using equation (2). Subsequently, these *R_dr_* are reduced synchronously across all ranks for the normalization operation of equation (3). The resultant normalized *P_dr_* array is used to calculate updated tomograms for each *r*, and then merged to obtain an updated three-dimensional model for each MPI rank. These models are finally reduced to obtain an updated model *W*′.

In many experimental situations, the incident fluence varies between X-ray pulses. Thus, the tomograms would be scaled differently for each pattern (Loh *et al.*, 2010 ▸; Ekeberg *et al.*, 2015 ▸). One can enable the recovery of these scale factors using the update rule described in Appendix *C* [App appc].

We also find that, if the signal on each pattern is too strong, and when the rotation group sampling is too fine or the data are too few, reconstructions can get stuck in a local maximum in which all frames are assigned to far too few orientations in reciprocal space. This effect is similar to what is observed if the background is too high (Ayyer, Geloni *et al.*, 2015 ▸). However, such reconstructions are empirically stable around the true solution, *W* ^true^, and only get trapped when one starts from random initial guesses. This problem can be avoided by using the deterministic annealing variant of expectation maximization (Ueda & Nakano, 1998 ▸). In the EMC case, this is implemented by raising *R_dr_* calculated in equation (2) to a small power β and then normalizing as in equation (3): *P_dr_* = 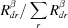. Doing this has the effect of broadening the orientation distribution and results in a rotationally blurred but stable reconstruction. Once the intensities of a metastable model have been resolved, the power β can then be raised gradually in a manner similar to simulated annealing, to guide the reconstruction slowly to the true global maximum around *W* ^true^. An example of this is shown in §3.2.2 and elaborated in Appendix *F* [App appf].

### 2.4. Software modules and convenience utilities   

The modules and utilities here are written in the programming languages C or Python (files with *.py extensions). For the system requirements to run the code, see §5.

#### 2.4.1. Simulation modules of data-stream generator   

Here we list the essential modules for simulating a data stream from an SPI experiment. By default, these modules use parameters listed in a single config.ini configuration file (detailed in §2.5.1), although different modules can use different configuration files as well. These modules can be either executed by the user or invoked by the convenience utilities described in §2.4.3. Users attempting the former are encouraged to study how these convenience utilities call the underlying modules.

(i) make_detector.py. Creates a detector file using the experimental parameters specified in the configuration file. The format of this file is specified in §2.5.2.

(ii) make_densities.py. Creates an electron-density map from an atomic model in the Protein Data Bank (PDB) format, given the resolution and field of view calculated from the configuration file. A low-pass filter is applied to this electron-density map to effect the intensity fall-off of atomic form factors.

(ii) make_intensities.py. Creates a set of three-dimensional diffraction intensities from an electron-density map and the experimental parameters found in the configuration file.

(iv) make_data. Simulates a sparse photon diffraction pattern using a three-dimensional diffraction volume (*e.g.* the one generated by make_intensities.py) and the configuration file. By default these photon data are saved as a binary file, photons.emc, detailed in §2.5.3. One can include a pattern-wise Gaussian spread in the incident fluence on the particle, as well as a uniform background.

#### 2.4.2. The EMC executable   

This executable reconstructs a three-dimensional diffraction volume from SPI data and is at the heart of the *Dragonfly* package. From Figs. 2 ▸ and 3 ▸, we see that data from either simulation or experiment workflows all converge into this EMC executable.

Internally, EMC creates a list of quasi-uniform rotation-group samples based on a refinement scheme of the 600 cell, detailed in Appendix *C* of Loh & Elser (2009 ▸). This level of refinement is defined by the num_div parameter in config.ini.

This EMC executable is implemented in the programming language C, using both the MPI and OpenMP parallelization frameworks. This hybrid implementation means that the user could choose to activate either or both types of parallelization. For example, one could run five iterations of a single-threaded single-process reconstruction using the command ./emc -t 1 5; omitting the -t 1 option uses the maximum available number of threads under OpenMP, typically specified by the shell variable OMP_NUM_THREADS. For a pure MPI version with 16 processes on the same node, the command is mpirun -np 16 ./emc -t 1 5. Finally, to run a hybrid version with the maximum available number of threads on six nodes, the command is mpirun -np 6 -H <hostnames> ./emc 5, where <hostnames> is a comma-separated list of node names on which the MPI process would run. Note that with OpenMPI 1.7+ one should use the --bind-to none option to make sure all cores in a thread are used. Different bindings may be available on different architectures.

#### 2.4.3. Convenience utilities   

Several convenience utilities are included to help prepare the data for or to view the results from the EMC reconstruction algorithm. The functions of these utilities, which are non-essential for the reconstruction and can be easily substituted, are briefly described here.

(i) init_new_recon.py. This Python utility compiles the C executables in the package, and makes them and the rest of the utilities available in a newly initialized reconstruction subdirectory. This utility calls the included Makefile that users can modify to customize this compilation.

(ii) sim_setup.py. This Python utility simulates an SPI data stream and calls modules listed in §2.4.1 – make_densities.py, make_intensities.py and make_data.py – subject to the configuration file parameters listed in §2.5.1.

(iii) make_powder.py. Makes a virtual powder pattern from all the diffraction patterns stored in the sparse photon format described in §2.5.3.

(iv) run_emc.py. Starts the EMC reconstruction by calling the EMC executable (see §2.4.2). Includes a few convenience operations, like increasing the sampling of the rotation group and/or continuing from a previous reconstruction.

(v) autoplot.py. Renders the results of the EMC reconstruction, including the diagnostics it generates, with the option of automatically updating the plots when newer intensities become available.

(vi) frameviewer.py. Viewer utility that plots the individual sparse photon files stored in the EMC format as they were measured on a planar detector (see §2.5.3).

### 2.5. Input and output to *Dragonfly*   

Here we specify only the input and output for the experimental workflow outlined in Fig. 3 ▸. The formats for the data-stream generator workflow in Fig. 2 ▸ are auxiliary to the reconstruction algorithm and are only detailed in the distributed software package.

#### 2.5.1. Configuration file   

**Figure 4:**
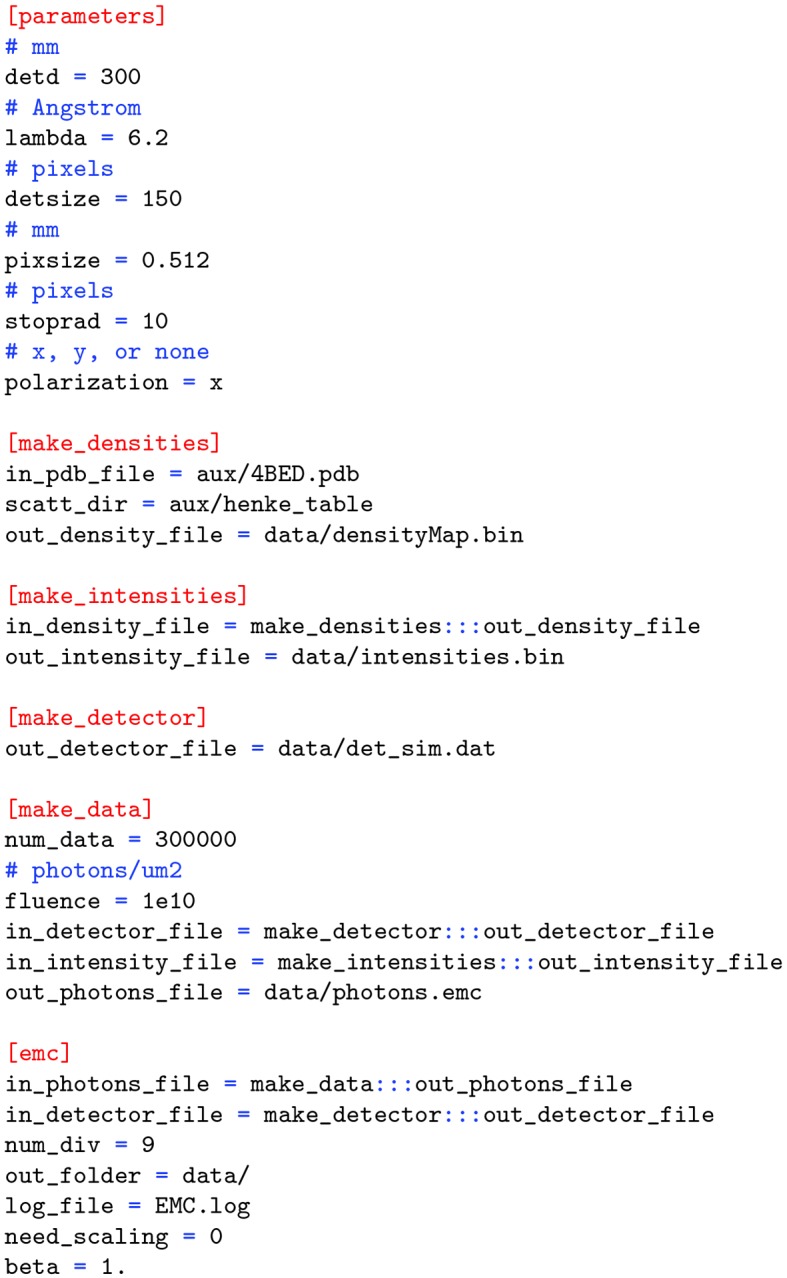
A typical configuration file, describing various parameters used to perform a basic simulation and reconstruction using the KLH1 (4BED.pdb) molecule on the AMO beamline. These parameters are to be compared with the numbers in Table 1 ▸.

The plain-text configuration file contains parameters and file names to be used by the EMC reconstruction as well as by the various modules/utilities. The file has the standard key = value format, with the parameters for different modules grouped by module names in square brackets. There is a global [parameters] section containing information about the experimental setup. A typical configuration file is shown in Fig. 4 ▸, which corresponds to the first simulation case in Table 1 ▸. This default file also shows the use of special keywords used to point to other configuration-file parameters (*e.g.* in_photons_file). The [parameters] section is described below. For other sections, refer to the appropriate module in §2.4.

The basic parameters of the experiment are as follows. Note that the fields with asterisks are only used in the data-stream simulator.

(i) detd: detector distance in mm.

(ii) lambda: wavelength in Å.

(iii) detsize^*^: detector size (assuming a square detector) in pixels.

(iv) pixsize: pixel size in mm.

(v) stoprad^*^: radius of the beamstop in pixels.

(vi) polarization: polarization direction of the X-ray pulses (can be x, y or none).

#### 2.5.2. Detector file   

The detector file is an ASCII (human readable) file which describes various properties of the detector. This file can be generated by make_detector.py as described in §2.4.1, or manually elsewhere.

The first line of this detector file specifies the number of pixels. Subsequently, individual pixels are described by five columns of numbers, with one pixel per line. The first three columns give the three-dimensional coordinates of the detector pixel in voxel units, where the voxels refer to the three-dimensional grid containing the intensity model. The mapping of detector pixels to spatial frequencies is described in Appendix *D* [App appd], and the pixel’s absolute size is specified by the pixsize field in the configuration file. The fourth column gives the product of the polarization (see polarization field in the configuration file) and solid-angle corrections for that pixel (Appendix *B* [App appb]). The last column is an eight-byte unsigned integer whose value is used by the EMC code and by other utilities to categorize the pixel. Currently, three pixel categories are implemented:

[0]: good pixels, used to determine the orientation of a given pattern and updated into the new intensity model.

[1]: these pixels will not be used to determine the orientation, but will still be merged into the three-dimensional grid using the orientations calculated from category 0 pixels. These are usually pixels in the corners of the detector.

[2]: bad pixels, which are either dead pixels or pixels within the beamstop. Their values will be used neither to determine the orientation nor to calculate the merged three-dimensional intensities.

Multiplying a data frame by these pixel categories removes good pixels, thus ‘masking them out’. Pixel categorization provides flexibility to users. For example, the beamstop(s) or gaps in the planar detector could be entirely omitted in a detector file that only contains the locations of good pixels. Alternatively, beamstop/gap pixels could be labeled ‘bad’ (category 2) if one uses a packed rectilinear set of pixel positions. This second approach allows the user readily to revise the pixel categories in an existing detector file.

Although the pixels from the data-stream simulation included here correspond to a dense planar detector (Fig. 1 ▸), these pixel locations can be arbitrary. However, a rule of thumb for SPI is that the locations of these pixels, though arbitrary, should evenly populate a contiguous range of scalar spatial frequencies up to the desired resolution. This way, sufficiently many patterns that are oriented uniformly in orientation space and measured on these pixels should densely fill the desired three-dimensional diffraction volume.

Finally, we emphasize that the ‘spatial frequency lookup table’ format of this detector file is convenient for compound detectors with gaps or missing tiles, or comprising tiles placed at different distances from the sample. In these cases, a special geometry consideration becomes unnecessary once the pixels on these non-contiguous detectors have been mapped onto the Ewald sphere in the detector file. Mapping to spatial frequencies in detector.dat is straightforward if a pixel location lookup table similar to that used by Barty *et al.* (2014 ▸) is available.

#### 2.5.3. Photon file (EMC format)   

Since the photon data in many high-resolution SPI experiments expect few photons per pattern, a sparse binary format is used to store the data. Hence, for each pattern we only store information about pixels that receive photons. Additionally, since most of the non-zero counts are ones, only their pixel locations are stored. For pixels receiving two or more photons, we store both their pixel location and their photon count.

**Figure 5:**
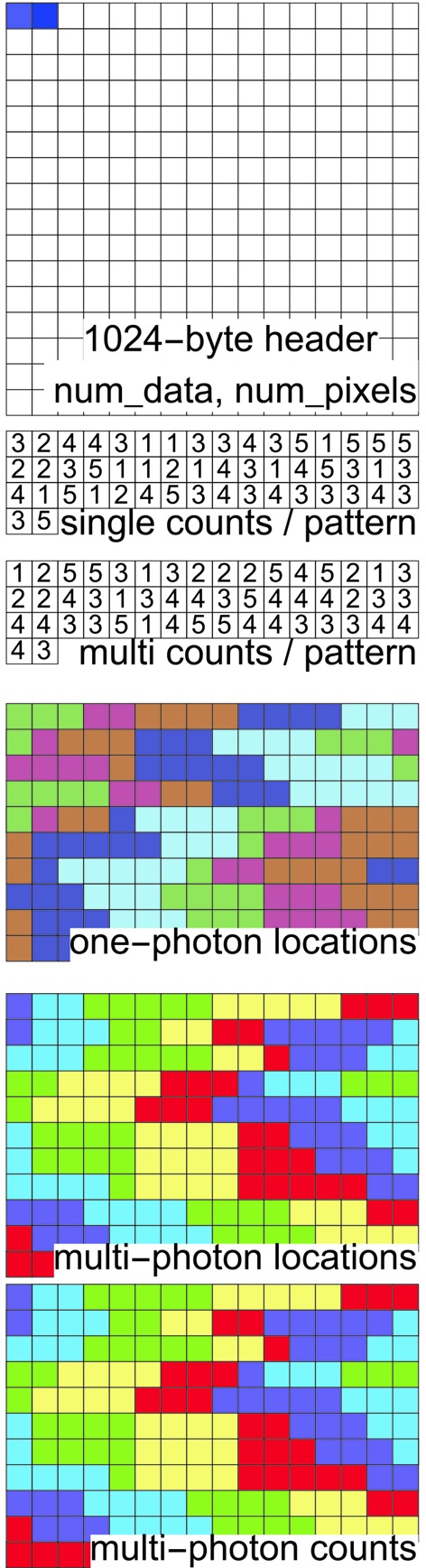
Six blocks in the sparse binary data format for 50 patterns. The data are stored contiguously but shown here in row-major format (*i.e.* to be read from left to right, then down the rows). Each square represents a 32-bit integer. The two integers in the header block are the number of patterns, followed by the number of pixels in the detector. The colors in blocks three to six connect listings of the same pattern. Details given in §2.5.3.

The data in the photon file are arranged in six blocks (Fig. 5 ▸). The file’s header resides in the first block, which is 1024 bytes long. This begins with two four-byte chunks: a 32-bit integer describing the number of patterns (num_data) contained in the file, followed by another 32-bit integer for the number of pixels in each pattern. These pixels include all three pixel categories stated in §2.5.2. The next 1016 bytes are currently unused and are filled with zeros.

The second block contains num_data 32-bit integers giving the number of one-photon events in each pattern (ones). The third block contains num_data integers giving the number of multi-photon events (multi). The total number of single-photon events in all the patterns is the sum of all the numbers in the ones array (*S*_o_). Similarly, let *S*_m_ be the total number of multiple-photon events. The fourth block contains *S*_o_ 32-bit integers giving the locations of the single-photon pixels. The fifth block has *S*_m_ integers with the locations of the multiple-photon pixels. Finally, the sixth block has *S*_m_ 32-bit integers giving the number of photons in each of those multiple-photon pixels.

To become familiar with this EMC photon format, the reader is encouraged to examine the frameviewer.py utility in this package (listed in §2.4.3) and its output.

#### 2.5.4. Output intensities   

The output three-dimensional intensities from the EMC executable in the workflows of Figs. 2 ▸ and 3 ▸ are saved as dense row-major order binary native-endian files (64-bit floating point), numbered according to the iteration number in the reconstruction. When one restarts a previous EMC reconstruction, the EMC executable will assume that the last iteration was the largest numbering suffixed on the file names of these intensities.

## 3. Example reconstructions of simulated experiments   

The use of the *Dragonfly* package is exemplified in three simulated SPI reconstructions using the specifications of the atomic, molecular and optical science (AMO) (Ferguson *et al.*, 2015 ▸) and coherent X-ray imaging (CXI) (Liang *et al.*, 2015 ▸) endstations at the LCLS (Emma *et al.*, 2010 ▸). We chose to simulate SPI of the keyhole limpet haemocyanin 1 (KLH1) didecamer (Gatsogiannis & Markl, 2009 ▸) and four-layer tobacco mosaic virus (TMV) (Bhyravbhatla *et al.*, 1998 ▸) on the AMO and CXI beamlines, respectively. It is notable that the choices in Table 1 ▸ yield an average of about 100 photons per single-particle diffraction pattern (pixel categories 0 and 1). Pattern-to-pattern intensity scaling was turned off in both data simulations and reconstructions.

The simulation parameters are shown in Table 1 ▸. The detectors here have 150 × 150 pixels, with the pixel sizes chosen to emulate a 1024 × 1024 pixel pnCCD (Strüder *et al.*, 2010 ▸) and CSPAD (Philipp *et al.*, 2011 ▸; Hart *et al.*, 2012 ▸). We decreased the beam fluence to obtain mean photon counts *N* ≃ 90 (the sum of pixel categories 0 and 1) for the first two simulations, mimicking realistic losses from imperfect beam transmission, optics and cleanup apertures (Loh *et al.*, 2013 ▸). The third simulation of Table 1 ▸ was designed to demonstrate how deterministic annealing can deal with the convergence issues caused by a very high signal (see end of §2.3 and Appendix *F*2). In this case, most of the parameters were identical to the low-fluence AMO case, except the fluence was up-adjusted to receive 1 mJ X-ray pulses, which is within an order of magnitude of the design specifications (Emma *et al.*, 2010 ▸).

For data sufficiency we use the signal-to-noise ratio parameter defined in equation (37) of Loh & Elser (2009 ▸), 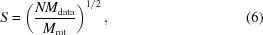
to estimate the required number of data frames *M*_data_ for *S* ≃ 50, where *M*_rot_ is the number of quasi-uniform rotation samples and *N* is the mean photon count per pattern. Assuming the diffraction patterns are uniformly distributed in orientation space, *S* ^2^ can be interpreted as the average number of photons per orientation.

### 3.1. Diagnostics on simulated reconstructions   

**Figure 6:**
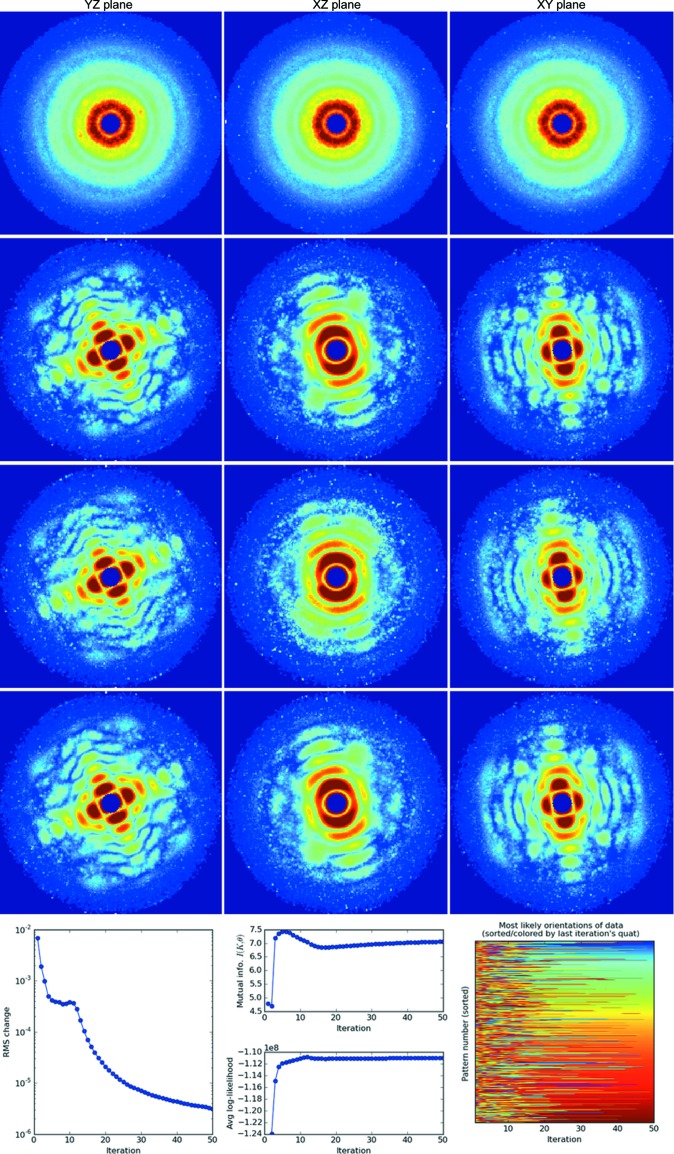
Convergence of diffraction speckle features in a simulated AMO single-particle experiment (parameters listed in Table 1 ▸). In each row we render central slices of the three-dimensional diffraction intensities recovered from KLH1 during an EMC reconstruction, after one, ten, 20 and 50 iterations in ascending row order. (Bottom row) Additional diagnostics on the reconstructed three-dimensional diffraction model. (Left) The r.m.s. change in the three-dimensional model. (Middle) Mutual information and log-likelihood of the model. (Right) The most likely orientations of all the patterns.

In this section, we describe useful diagnostics for monitoring the progress of each three-dimensional reconstruction. Figs. 6 ▸, 7 ▸ and 8 ▸ show orthogonal slices through the reconstructed intensities for the three parameter sets in Table 1 ▸. Below each figure is a set of plots generated by the autoplot.py utility, which helps to visualize these diagnostics.

We discuss these diagnostics starting with the AMO reconstruction in Fig. 6 ▸, which consistently converges from random restarts. With each new reconstruction attempt, diffraction speckles converge readily, although each time at a different overall orientation.

#### 3.1.1. R.m.s. change in the three-dimensional model   

The root mean-squared (r.m.s.) change per voxel between the three-dimensional intensity models from successive iterations in Fig. 6 ▸ is a straightforward indicator of convergence. Model changes decrease as the algorithm converges. Note that a converged model might not always be the solution (see Appendix *F*1).

#### 3.1.2. Mutual information between model tomograms and data   

An additional diagnostic is the ‘sharpness’ of the probability distribution over orientations *P*_*dr*_ calculated in equation (3), which one expects to increase as a reconstructed model converges. This sharpness can be monitored from the mutual information of the joint distribution of the data and the orientations, *P*(*K*, Ω) = *P*(Ω)*P*(*K* | Ω) = *P_r_* *P_dr_*. Here, *P_r_* is the prior probability of orientations *r* (assumed here to be uniform). The mutual information between the data and the tomogram of orientation Ω given the current model *W* is evaluated as 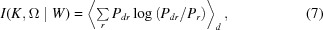
where 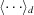 is the average over data frames. Equation (7) approaches the entropy of the rotation-group sampling when each pattern fits only one orientation, while it vanishes for a uniform distribution.

The fact that this mutual information rises asymptotically in Fig. 6 ▸ (center of bottom panel, top plot) is consistent with model convergence. Below it is a plot of the model log-likelihood defined in equation (4). This quantity should increase monotonically as the three-dimensional model eventually converges, although transiently high values may be consistent with EMC over-fitting patterns to low-likelihood early models (see Figs. 6 ▸ and 7 ▸).

#### 3.1.3. Average log-likelihood of patterns given a model   

The previous two diagnostics largely indicate if a model’s reconstruction has converged and offer less information about whether the model is ‘likely’. Here we introduce a third diagnostic, the log-likelihood of all the data patterns given the current three-dimensional model, as computed in equation (4). This likelihood quantifies how an iterative reconstruction approaches a global likelihood maximum. This diagnostic is plotted in the bottom panel of the middle columns in Figs. 6 ▸, 7 ▸ and 8 ▸. Again, note that a likely model might not always be the true solution (see Appendix *F*1).

#### 3.1.4. Most likely orientations of each pattern   

We describe the most detail-rich and possibly revealing diagnostic for monitoring convergence. Consider the matrix plot in the bottom rightmost panel of Fig. 6 ▸: its vertical axis labels the pattern number while the horizontal axis labels the iteration number. The color rendered represents the orientation number of the most likely orientation (maximum *P_dr_*) for each pattern. In Fig. 6 ▸ we sorted the patterns by the most likely quaternion in the final iteration of each block having the same rotation-group sampling. As a result, the colors at the right-hand end form a smooth spectrum and the pattern numbers differ between rotation-sampling blocks. The variation in color along a row indicates how the most likely orientation has changed for that pattern. The patterns settle into their most likely orientations when these colors become constant with iteration, which is a good indicator of convergence.

**Figure 7:**
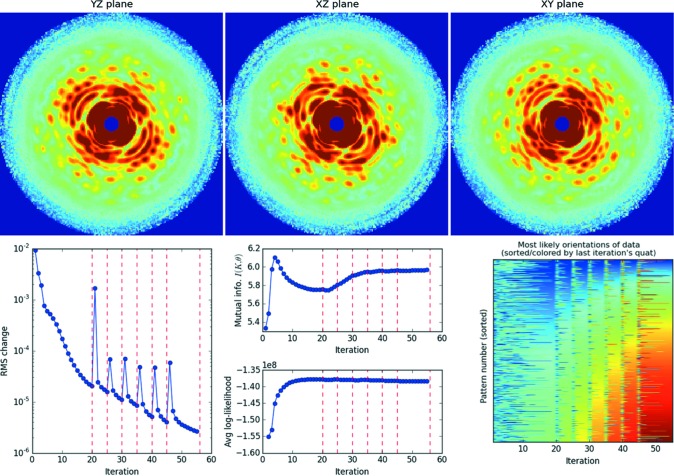
Rotation-group refinement for a simulated reconstruction of TMV on the CXI endstation (see Table 1 ▸). Shown here are the central sections of the reconstructed three-dimensional diffraction volume of TMV after 55 iterations. With 90 Intel Xeon X7542 (2.67 GHz) cores, this full reconstruction took less than 6 h, taking 15 min for each of the slowest refinement iterations using 204 960 rotation-group samples. Red dashed lines in the r.m.s. model change mark when the refinement level of the rotation group was increased by one. In the bottom right-hand plot, rows are colored by each photon pattern’s most likely orientation number, which stabilizes after 20 iterations and thereafter quickly re-stabilizes when we increase the rotation-group refinement. The rows (pattern indices) are sorted according to the most likely orientation indices of the last iteration in each rotation-sampling block, which produces a smooth color spectrum along this final column. Since the number of quaternions (quat) increases with rotation refinement, blocks of higher refinement show a wider color spectrum. See §3.2.1 for details.

This diagnostic is also useful for cases when the rotation-group sampling is increased steadily (*i.e.* Fig. 7 ▸). For each iteration block where the rotation-group sampling is fixed, we sort the patterns (rows in this orientation plot) such that the last iteration in the block has ascending orientation numbers. However, whereas the pattern index is constant within each block, they differ between rotation-group sampling blocks because each block is sorted separately.

### 3.2. Strategies for reconstruction   

#### 3.2.1. Gradually increasing rotation-group sampling   

For the CXI reconstruction (Fig. 7 ▸), owing to the size of the problem, the quaternion sampling number was increased in steps. If one chooses too coarse a rotation-group sampling, low-resolution speckles are reconstructed but higher-resolution features remain blurry. These higher-resolution speckles sharpen quickly when we increase the rotation-group sampling for reconstructions starting from this blurry model. Since the computation time scales as the number of rotation-group samples, it is faster to increase rotation-group sampling gradually such that only a few iterations are performed with the most time consuming but finest sampling. Red dashed lines in the bottom plots of Fig. 7 ▸ indicate iterations where the rotation-group refinement level was increased gradually from ten to 16 (details in Table 1 ▸). Note that the mutual information does not increase much in the last rotation-group refinement, indicating that further refinement would not substantially improve the model likelihood. The Python utility run_emc.py listed in §2.4.3 has a -R option for increasing the level of rotation-group sampling of a reconstruction by one. In general, we found good results when manually increasing this sampling, once the changes in speckle features have visibly converged.

#### 3.2.2. Regularization *via* deterministic annealing   

**Figure 8:**
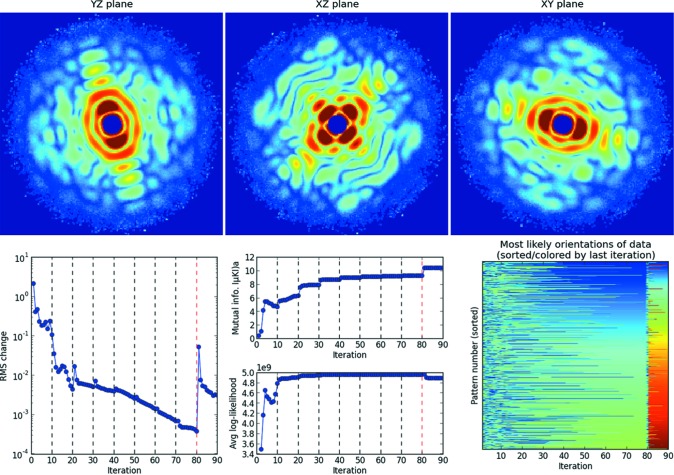
Deterministic annealing in a simulated reconstruction on the AMO endstation with high photon fluence (see Table 1 ▸). This reconstruction was performed by doubling the β parameter (§3.2.2) every ten iterations, starting from β = 0.001. Doublings occur at the dashed black lines in the diagnostic plots in the bottom row, where the ten-iteration interval was chosen to allow the intermediate reconstructions to stabilize. This stabilization can be judged by the asymptotic saturation of the average mutual information in every β block. After 80 iterations (β = 0.256), this increase was stopped as there did not seem to be much further improvement in the average mutual information. After this, the rotational sampling rate was increased from six to the target of nine. As in the CXI reconstruction (Fig. 7 ▸), this was done in order to save computational time by doing fewer iterations at the highest sampling.

The high-fluence AMO reconstruction (Fig. 8 ▸) assembles patterns of very high signal-to-noise ratio. Hence, starting the algorithm from random initial models can cause the iterative reconstruction to behave erratically (see Appendix *F* [App appf]). This can be avoided by starting with a low β, as described in §2.3, which reduces the propensity for erratic updates between EMC iterations. In this particular case, β was 0.001 for the first ten iterations. Once this intermediate reconstruction converged, we gradually doubled β every ten iterations to restore the speckles to the highest contrast allowable by the data and likelihood model. The black dashed lines in Fig. 8 ▸ represent the iterations when β was doubled. After 80 iterations, the rotation-group refinement level was increased from six to nine, and continued for another ten iterations (see §3.2.1 for rotation-group refinement). It is evident in Fig. 8 ▸ that the speckle features in the reconstructed intensities sharpen when β rises back near unity.

In the software package, one can either increase β manually after a few iterations and restart the reconstruction, or use the hidden option beta_schedule in config.ini. This second option takes two whitespace-separated numbers, beta_jump and beta_period; β is multiplied by a factor of beta_jump every beta_period iterations.

## 4. Conclusions and future work   

Future work can be divided broadly into the two main use cases, namely simulations and experimental data. For simulations, we plan to include support for non-uniform background distributions, both for data generation and to be used as *a priori* knowledge during the reconstruction. We also plan to include realistic distributions for incident fluence fluctuations. One significant challenge in single-molecule imaging is the heterogeneity of the particles between patterns. For particles with a few conformation classes, one can reconstruct multiple three-dimensional model intensities simultaneously by solving for both the orientation and the class index (Loh, 2012 ▸). We plan to implement this for both the data-generation pipeline and the EMC code.

To deal with experimental data, we will add utilities to convert current experimental data to the sparse emc format. Similar utilities will be provided to generate detector files from a variety of formats currently employed to describe the experimental geometry. The ability to deal with a known structured background, mentioned above, would also be valuable for experimental data: the user would be able to provide a measured ‘background file’ to the reconstruction code. There are also plans to incorporate single-particle reconstruction while learning and rejecting an initially unknown background (Loh, 2014 ▸).

## 5. Access to EMC   

The source code for this software package can be downloaded from http://duaneloh.github.io/Dragonfly/ and is distributed under the terms of the GNU General Public License (GPL, Version 3; http://www.gnu.org/licenses/gpl). Instructions to run a basic simulation are available in the README file available with the repository. In addition, one can find detailed up-to-date documentation in the repository wiki accessible at http://github.com/duaneloh/Dragonfly/wiki. This wiki includes descriptions of all the options available for each of the modules and utilities supplied in the package.

The modules and utilities are written in C and Python 2.7. The C files require the following libraries to compile: *mpi*, *openmp* and the GNU Scientific Library (http://www.gnu.org/software/gsl). The Python files need Python version 2.7.x to run, and the non-standard libraries *NumPy* and *SciPy* (http://www.scipy.org).

## Figures and Tables

**Figure 9:**
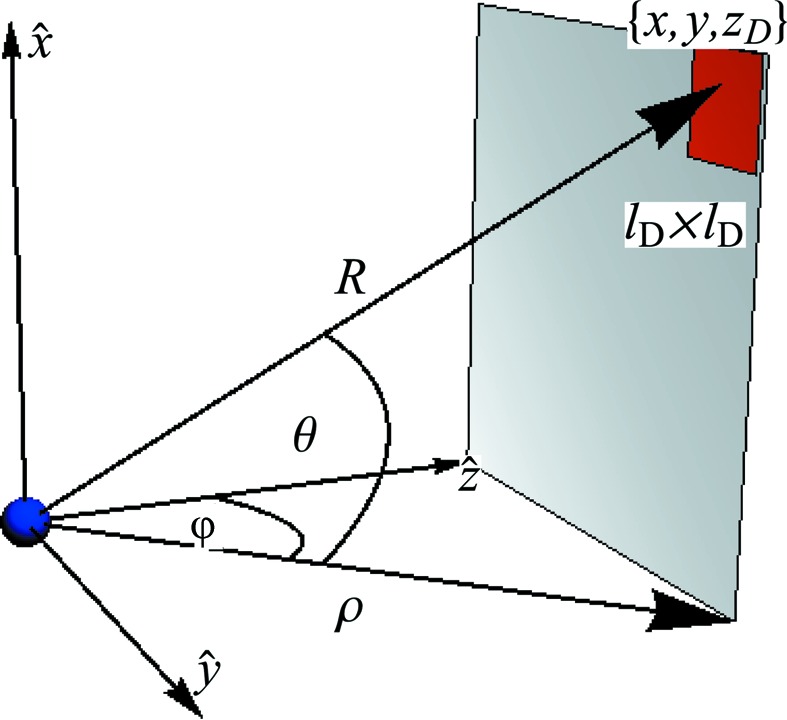
Setup for solid-angle correction. We compute the solid angle subtended by the square pixel (red) on the detector plane (grey). The scatterer (blue sphere) is set at the origin of this figure.

**Figure 10:**
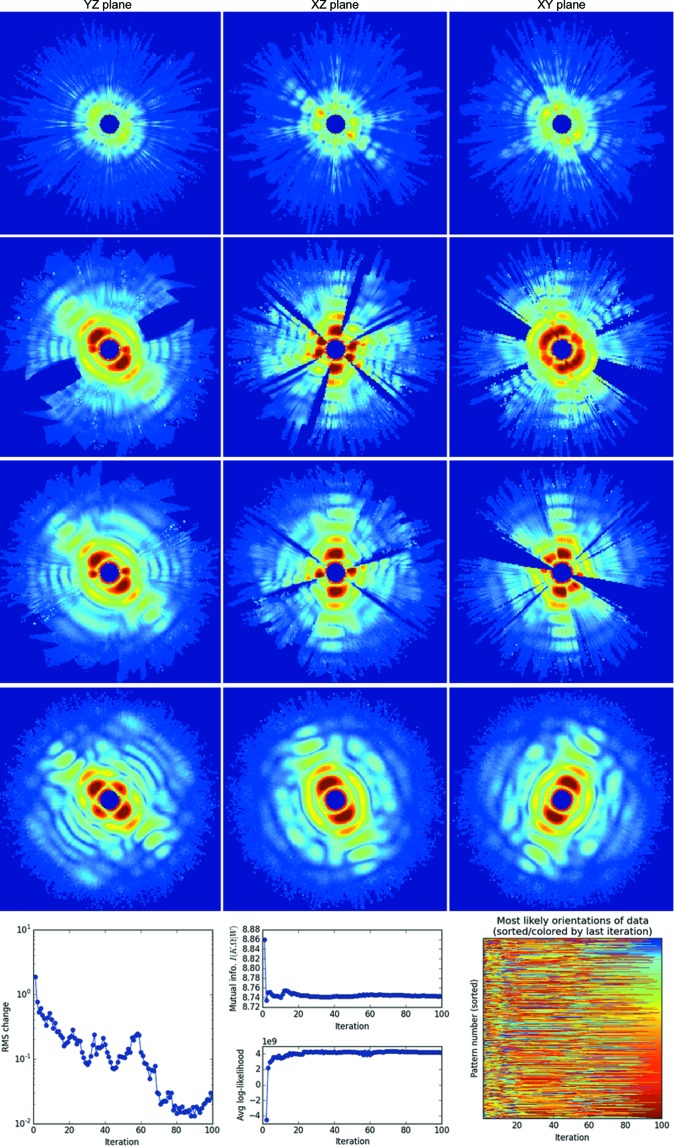
Low-intensity wedge-like volumes appear in the EMC-reconstructed volume with very high signal data frames (most frames contain more than 10^4^ photons). The simulation parameters are listed in Table 1 ▸ as AMO (high). We reconstructed with rotation-group refinement *n* = 5 and with β = 1 (annealing turned off). In descending order down the rows, the panels show the central sections of the updated model after one, five, ten and 100 iterations, and the lower panels show the diagnostics for 100 iterations (plots described in the caption to Fig. 6 ▸).

**Table 1:** Parameters for EMC reconstructions of simulated single-particle imaging

	AMO (low)	CXI	AMO (high)
Photon energy (keV)	2.0	7.0	2.0
λ, photon wavelength (Å)	6.2	1.77	6.2
*z*_D_, detector distance (mm)	300	350	290
*d*, detector size (pixel)	150	150	150
*l*_D_, pixel side length (mm)	0.512	0.751	0.512
*L*, full field of view (nm)	363	82.4	351
Beamstop radius (pixels)	10.0	8.0	10.0
Fluence (photons µm^−2^)	1 × 10^10^ †	1 × 10^12^	**3.1 × 10^12^**
*a*, half-period resolution‡ (nm)	2.45	0.56	2.5
Particle	KLH1§	TMV¶	KLH1§
Mass (MDa)	7.3	1.3	7.3
*R_p_*, particle radius (nm)	18.9	9.3	18.9
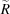 ††, dimensionless radius	7.7	16.6	7.6
σ, speckle sampling‡‡	9.6	4.45	9.2
*N*, mean photons per frame	90	90	**2.8 × 10^4^**
No. of data frames	3 × 10^5^	5 × 10^5^	1 × 10^5^
Max. quaternion sampling§§	9	16	9

† Estimated from Fig. 4 in the paper by Loh *et al.* (2013 ▸).

‡ Resolution defined from the detector edge.

§ Keyhole limpet haemocyanin 1.

¶ Four-layer tobacco mosaic virus.

†† Dimensionless radius, *R_p_*/*a*.

‡‡ Defined as 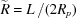. See Appendix *A* [App appa].

§§ The sampling and criterion are defined in Appendix *C* [App appc] and Section VII of Loh & Elser (2009 ▸), respectively.

**Table 2:** How the sizes of parameters in reconstructions scale with a particle’s dimensionless resolution 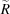

Parameter	Scales as
No. of detector pixels†, *M*_tomo_	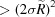
No. of rotation-group samples‡, *M*_rot_	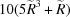
No. of data frames[Table-fn tfn10], *M*_data_	*S* ^2^ *M*_rot_/*N* = 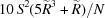
No. of conditional probabilities, *M*_prob_	*M*_data_ *M*_rot_ = 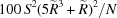
No. of model voxels per MPI process[Table-fn tfn11], *M_W_*	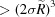
No. of sparse data entries per MPI process[Table-fn tfn12], *M*_sp_	≤*NM*_data_

† The speckle sampling, σ, is defined in Appendix *A* [App appa].

‡ The sampling and criterion are defined in Appendix *C* [App appc] and Section VII of Loh & Elser (2009 ▸), respectively.

§ Signal-to-noise ratio, *S*, defined in equation (6).

¶ Model represented as a dense cubic array.

†† See sparse data format in §2.5.3.

**Table 3:** Memory requirements for modest-fidelity reconstruction: *S* = 10, *N* = 100 and σ = 5 The different variables are explained in Table 2 ▸.

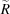	*M*_data_	*M*_rot_	*M*_sp_ (MB)	*M*_*W*_ (MB)	*M*_prob_ (GB)
5	6300	6300	4.81	0.953	0.296
10	50100	50100	38.2	7.63	18.7
15	168900	168900	129	25.7	213
20	400200	400200	305	61.0	1193
25	781500	781500	596	119	4550
30	1.35 × 10^6^	1.35 × 10^6^	1030	206	13584
35	2.14 × 10^6^	2.14 × 10^6^	1635	327	34251
40	3.20 × 10^6^	3.20 × 10^6^	2441	488	76313
45	4.56 × 10^6^	4.56 × 10^6^	3476	695	154700

**Table 4:** Memory requirements for high-fidelity reconstruction: *S* = 50, *N* = 100 and σ = 5 The different variables are explained in Table 2 ▸.

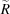	*M*_data_	*M*_rot_	*M*_sp_ (MB)	*M*_*W*_ (MB)	*M*_prob_ (GB)
5	157500	6300	120	0.953	7.39
10	1.25 × 10^6^	50100	956	7.63	468
15	4.22 × 10^6^	168900	3220	25.7	5310
20	1.00 × 10^7^	400200	7630	61.0	29800
25	1.95 × 10^7^	781500	14900	119	114000
30	3.38 × 10^7^	1.35 × 10^6^	25800	205	340000
35	5.36 × 10^7^	2.14 × 10^6^	40900	327	856000
40	8.00 × 10^7^	3.20 × 10^6^	61000	488	1.91 × 10^6^
45	1.14 × 10^8^	4.56 × 10^6^	86900	695	3.87 × 10^6^

## Appendix A Computing speckle sampling on the detector

We use a spherical approximation to estimate the size of diffraction speckles from a scatterer. A sphere of radius *R_p_* produces diffraction intensities

with dimensionless spatial resolution , where we define  = 2sin(φ/2)/λ as the spatial frequency commonly used in structural biology. Here, φ and λ are the scattering angle and photon wavelength, respectively (see Fig. 1 ▸). The width of a diffraction speckle of this spherical approximation is the separation in  between the zeros of equation (8). These zeros occur when

which approaches  (2*n* + 1)π/2, where , for large . As a result, for large  the separation between the zeros of equation (8) , which results in a speckle width

Referring to Fig. 1 ▸, the coarsest spacings between the spatial frequency samples occur at small scattering angles and are inversely proportional to the field of view *L*, or  ≃ 1/*L*.

The sampling ratio of the diffraction speckle is defined as

where *l*_D_ is the width of the detector pixel and *z*_D_ is the separation between the detector and the interaction region (see Figs. 1 ▸ or 9 ▸). While the ideal sampling ratio of the diffraction speckles should exceed two, the time and memory required for intensity reconstruction rises rapidly when this ratio becomes excessively large (*e.g.*  ≫ 5).

## Appendix B Solid-angle and polarization correction for square pixels on planar detectors

### b1. Solid-angle correction

In this section we compute the solid angle ΔΩ subtended by a square pixel of area *l*_D_ × *l*_D_ about the point where a scatterer sits (see Fig. 9 ▸). To a first approximation, the solid angle ΔΩ ≃ cos(θ)ΔφΔθ. We use the following relations to estimate Δφ and Δθ. On a detector *z*_D_ from the interaction point, the spherical coordinate representation of a pixel at Cartesian coodinate {*x*, *y*, *z*_D_} is

where ρ = (*y* ^2^ + )^1/2^. Differentiating sin(φ) with respect to φ gives

Repeating this for θ, where

with *R* = (*x* ^2^ + *y* ^2^ + )^1/2^, leads to

Combining the two and simplifying, we obtain the solid angle subtended by the square pixel as

### b2. Polarization correction

Consider the case where the incident beam is polarized along the  = {1, 0, 0} direction in Fig. 9 ▸. This polarization reduces the scattered intensity by a factor *P* = 1 − |(*x*, *y*, *z*_D_)|^2^, where  is the unit-norm vector from the scatterer (placed at {0, 0, 0}) to the pixel located at {*x*, *y*, *z*_D_}. Notice that we can also write *P* = cos^2^θ, or entirely in the terms of the pixel’s coordinates

Similarly, when this polarization is along the  direction, the intensity reduction due to polarization becomes

## Appendix C Pattern-wise intensity scale factor updates

In many real-world applications, the incident fluence on each particle will be different. The original implementation of EMC in the paper by Loh & Elser (2009 ▸) assumes uniform incident fluence. Here, we derive the likelihood maximizing update rule employed in this package when the need_scaling option is turned on. The approach used is similar to that employed by Loh *et al.* (2010 ▸), except here we use a Poisson rather than a Gaussian probability model. Let φ_*d*_ be a scale factor which is proportional to the fluence incident on the particle in pattern *d*. Thus, equations (3) and (2) become

and

In expectation maximization, one updates the intensity tomograms, *W*′, and fluence, φ′, by maximizing the model log-likelihood:

Here, *P_dr_* are the probabilities calculated using the current models *W* and φ. Unfortunately, an analytical update rule that simultaneously updates both these quantities is not available. Instead, we use the strategy of updating one while keeping the other constant. Setting partial derivatives with respect to *W*′ and φ′ equal to zero, we obtain

This modification to the update rule in equation (5) is used when the user expects variable incident fluence on the particle.

## Appendix D Mapping the detector onto the Ewald sphere

We outline how pixels depicted in Fig. 9 ▸ are mapped onto the Ewald sphere during elastic scattering. Suppose the incident X-ray beam travels along the  direction, comprising photons of wavelength λ. This direct unscattered beam has a reciprocal vector (crystallographers’ convention)

Now consider a pixel on the detector whose center is {*x*, *y*, *z*_D_} away from the scatterer in the laboratory frame. When a photon is elastically scattered by the scatterer to this pixel, the photon has an approximate (because of the finite size of the pixel) reciprocal vector

Hence, this pixel measures the kinematic diffraction intensities of the scatterer at a spatial frequency

As a consequence of equation (26), pixels on a planar detector are mapped onto a curved surface known as the Ewald sphere, and this intersects the scatterer’s zero spatial frequency. The influence of this Ewald sphere curvature becomes more prominent as the sample-to-detector distance *z*_D_ is reduced.

The mapping in equation (26) applies to any arbitrary set of pixels, each with their own {*x*, *y*, *z*_D_} values, even if they span several non-contiguous detector tiles with custom gaps or missing regions. In general, the EMC algorithm permits such an arbitrary collection of pixels as long as they are properly specified in the detector.dat file. Although three-dimensional iterative phase retrieval may suffer from these missing pixels in the desired spatial frequency range, they do not affect the intensity assembly *via* EMC.

Finally, the highlighted pixel in Fig. 9 ▸ could be a collection of pixels binned as an effective ‘super-pixel’. This provision is useful when working with experimental data collected with overly redundant speckle oversampling. Here, down-sampling or binning pixels to create larger ‘super-pixels’ can be computer- and memory-efficient for EMC. However, the user should be aware that over-binning can result in a blurred real-space contrast from phase retrieval.

## Appendix E Memory requirements

In this section we estimate the amount of random access memory (RAM) needed for EMC reconstructions of various sizes and signal levels. Note that, because of implementation differences, the requirements of this software package differ from those originally described by Loh & Elser (2009 ▸).

From Table 2 ▸, we see that the important size scales in a reconstruction can be expressed in powers of , the dimensionless resolution of the recovered particle (defined in Appendix *A* [App appa]). Naturally, more memory is needed when reconstructing to higher dimensionless resolutions. Equivalently, the ‘speckle complexity’ of such reconstructions in reciprocal space also increases with resolution (compare Figs. 6 ▸ and 7 ▸).

From Table 2 ▸, note that the number of conditional probabilities computed by EMC scales like *M*_prob_ ≃ , which grows the fastest of all the parameter size factors. In the examples listed in Tables 3 ▸ and 4 ▸, the memory needed to store the sparse frames and the three-dimensional model *W* is many megabytes (MB) per MPI process. However, many gigabytes (GB) to terabytes (TB) of memory are needed to store the conditional probabilities *P_dr_* computed by EMC. Currently, this package stores these probabilities in RAM, but future versions may write them to disk if necessary.

## Appendix F Orientation instability at high signal levels

The EMC algorithm is capable of searching for three-dimensional diffraction volumes that maximize the likelihood of measuring a set of data frames. The performance of this algorithm is influenced by two considerations. The first is the ‘likelihood landscape’ of this problem: whether there are locally maximal false solutions that can trap the EMC search, and whether the family of true solutions are also global maxima. The second consideration is search dynamics: the time needed to reach these likelihood maxima, and the meta-stability of the local maxima. Although we cannot anticipate how these two considerations will affect all EMC reconstructions, this section gives a flavor of their importance.

### f1. Overfitting from having too few high-signal patterns

Here we consider a case when the global maximum is not the true solution. Suppose only two high-signal two-dimensional patterns are measured, {*A*_*t*_} and {*B_t_*}, where the index *t* labels the *M*_pix_ detector pixels of each pattern. We further assume that each pattern is diffracted from separate copies of the three-dimensional object at exactly the same orientation in the laboratory frame. However, despite their identical orientations, these two patterns will be quantitatively different because they are different noisy realizations of the same two-dimensional pattern. Two types of converged outcome are possible:

(i) place the two two-dimensional patterns at the same orientation and average over them, or

(ii) place them at two distinct orientations, such that the patterns only intersect along a one-dimensional ‘common arc’ in the three-dimensional volume.

To simplify case (i), we assume that both patterns adopt the same orientation. Hence, the log-likelihood of measuring {*B_t_*}, given that the underlying pattern with *M*_pix_ pixels averages over {*A_t_*} and {*B_t_*} under a Poissonian noise model, is

where the inequality arises because of Stirling’s approximation (log*n*! ≃ *n*log*n* − *n*, if *n* ≫ 1, and also log*n*! ≥ *n*log*n* − *n*) and approaches equality when *A_t_* ≫ 1 and *B_t_* ≫ 1 at high fluence. The complementary likelihood of  simply switches the *A* and *B* labels in equation (27), giving the combined log-likelihood of both patterns sharing the same orientation as

where α(*A_t_*, *B_t_*) denotes the expression inside curly brackets on the previous line. Using Gibbs’ inequality, , where equality occurs when *p_t_* = *q_t_*, and averaging over all possible values of *A* and *B*, we expect

Hence, the combined likelihood  in equation (28) must be negative and scales with the number of pixels *M*_pix_.

Case (ii) has a similar calculation, except that the pixels are separated into two categories: those that lie on the common line between the two oriented patterns, *M*_com_, and those that do not, denoted *M*_sep_ = *M*_pix_ − *M*_com_. Ignoring corrections due to interpolating these patterns into the three-dimensional volume, it is straightforward to show that the log-likelihood of measuring patterns {*A_t_*} and {*B_t_*} when they are placed in different orientations is

From equations (28), (29) and (30) it is clear that, on average

and

where *f* increases monotonically with the average photon count on the pixels |*A*| and |*B*|. Evidently, the incorrect ‘apart’ solution maximizes the likelihood here and belongs to the family of three-dimensional models that over-fit limited measured data. (Any well separated pair of orientations could be a maximum likelihood result as are, trivially, global rotations of these.)

### f2. Erratic EMC updates with many high-signal patterns

In this section, we describe why the iterative orientation discovery in EMC can be erratic when the signal levels from patterns are very high. This erratic behavior explains, in part, why the regularization routine in §3.2.2 was invoked.

The log-likelihood that a set of pixels on a pattern *K* was measured given a tomogram intensity *W*(Ω) can be decomposed into sums over zero and nonzero photon pixels:

This log-likelihood is never greater than zero. This is because 1 − *x* + log*x* ≤ 0, *K_t_* ≥ 0 and *W*(Ω)_*t*_ ≥ 0, since photon counts and the tomograms updated by equation (5) are each always positive.

For a pattern with a very high signal, the conditional probability distribution has a very narrow peak around the most likely orientation Ω^best^. From equation (33), the log-likelihood of *K* against a well matched orientation tomogram in the model, *W*(Ω), will yield modestly negative values for Σ_0_ and Σ_1_. This is because their photon and intensity values, {*K_t_*} and {*W*(Ω)_*t*_}, respectively, are also closely matched. However, when the signal levels on the data frames are high, a mismatched high-signal *K* and *W*(Ω) pair will give very negative log-likelihoods. As a result, the most likely *K* + *W*(Ω^best^) pair will be deemed very much more likely than pairings of *K* with any other *W*(Ω).

While a narrow orientation distribution for data frames is generally desired, the difficulty starts when the entire three-dimensional model *W* is far away from the true three-dimensional model. This scenario usually occurs during the first few iterations of an EMC reconstruction starting from a random initial *W*. Despite the erroneousness of *W*, the conditional probability of each data frame over the orientations will still have a narrow peak, for reasons similar to those presented above. Hence, the re-orientation of data frames within *W* by an EMC iteration happens resolutely and, in its wake, causes low-intensity wedge-like volumes to appear in the updated *W*′ (see Fig. 10 ▸), purely due to random fluctuations.

These low-intensity wedges form convergence traps for the algorithm. When *W* is far from the solution, its tomograms *W*(Ω) will be composed of randomly rotated and averaged subsets of the data frames. This results in very negative values of Σ_0_ in  for many frames *K*. Comparatively, any low-intensity wedges in the three-dimensional model contain sets of *W*(Ω^dark^) that yield much more positive Σ_0_ and will cause the *K* frames to be resolutely re-assigned to these Ω^dark^ orientations at the end of the EMC iteration. This resolute movement may in turn open up new low-intensity wedges in *W*. Further low-intensity wedges that do not resemble any *K* will ‘attract’ and result in yet another set of randomly rotated and averaged subsets of the data frames, paving the way for the next round of orientation re-assignment. This behavior results in erratic updates between EMC iterations, where low-intensity wedges will suddenly appear then disappear within a few iterations.

Incidentally, this erratic behavior should not occur when *W* is near the true solution, because data frames *K* then have no incentive to move away from their correctly determined orientations or create low-intensity wedges in *W*. Also, when the signal is lower, the orientation distributions are broader in the first few iterations, avoiding these wedges.

While enough random erratic updates may eventually yield the solution intensities, this iterative search can be relaxed using the β parameter in §3.2.2, which ‘spreads out’ these narrow peaks in the likelihood distributions. This way, low-intensity wedge-like volumes are less likely to appear in *W*′. Empirically, we note that this regularization can greatly improve the stability of the iterations towards the true intensities, even when the data frames have very high signal levels. Once *W* iteratively converges to the neighborhood of the true solution, then β can be raised back to 1.
